# Aberrant expression of *ALK* and *EZH2* in Merkel cell carcinoma

**DOI:** 10.1186/s12885-017-3233-5

**Published:** 2017-03-31

**Authors:** Tuukka Veija, Virve Koljonen, Tom Bohling, Mia Kero, Sakari Knuutila, Virinder Kaur Sarhadi

**Affiliations:** 1 Department of Pathology, Faculty of Medicine, University of Helsinki, Haartmaninkatu 3, P.O. Box 21, FI-00014 Helsinki, Finland; 2 Department of Pathology, University of Helsinki and HUSLAB, Helsinki University Hospital, Haartmaninkatu 3, P.O. Box 21, FI-00014 Helsinki, Finland; 3 Department of Plastic Surgery, University of Helsinki and Helsinki University Hospital, Topeliuksenkatu 5, P.O. Box 266, FI-00029 Helsinki, Finland

**Keywords:** Merkel cell carcinoma, Next-generation sequencing, RNA, ALK, EZH2

## Abstract

**Background:**

Distinct characteristic features categorize Merkel cell carcinoma (MCC) into two subgroups according to the Merkel cell polyomavirus infection. Many mutational studies on MCC have been carried out in recent years without identifying a prominent driver mutation. However, there is paucity reporting the expression of cancer genes at the RNA level in MCC tumors. In this study, we studied the RNA expression profiles of 26 MCC tumors, with a goal to identify prospective molecular targets that could improve the treatment strategies of MCC.

**Methods:**

RNA expression of 50 cancer-related genes in 26 MCC tumors was analyzed by targeted amplicon based next-generation sequencing using the Ion Torrent technology and the expression compared with that of normal, non-cancerous skin samples. Sequencing data were processed using Torrent Suite™ Software. Expression profiles of MCV-negative and MCV-positive tumors were compared. Fluorescence in situ hybridization was performed to study *ALK* rearrangements and immunohistochemistry to study ALK expression in tumor tissue.

**Results:**

*ALK, CDKN2A*, *EZH2* and *ERBB4* were overexpressed, and *EGFR*, *ERBB2*, *PDGFRA* and *FGFR1* were underexpressed in MCC tumors compared to normal skin. In the MCV-negative tumors, *MET, NOTCH1, FGFR3, and SMO* were overexpressed and *JAK3* and *NPM1* were under-expressed compared to the MCV-positive tumors.

**Conclusions:**

High expression of *ALK, CDKN2A and EZH2* was recorded in MCC tumors. No ALK fusion was seen by FISH analysis. Overexpression of *EZH2* suggests its potential as a drug target in MCC.

**Electronic supplementary material:**

The online version of this article (doi:10.1186/s12885-017-3233-5) contains supplementary material, which is available to authorized users.

## Background

Merkel cell carcinoma (MCC) is a neuroendocrine skin tumor with a high potential to metastasize. UV-radiation and Merkel cell polyomavirus (MCV) infection contribute to oncogenesis in MCC. [1] The viral oncogenic pathway accounts for the large majority of MCC tumors, as 80% of the tumors are MCV-positive. [2] Cumulative evidence suggests two MCC subgroups with and without MCV infection. [3–7].

Clonal integration of the MCV DNA into the tumor cell genome as well as mutations in the viral Large T Antigen enable oncogenic transformation in MCV-positive MCC. [2, 8] The non-viral pathway preceding MCV-negative MCC is less understood. Recent studies show that MCV-negative MCC tumors have a much higher mutational burden than MCV-positives. However, none of the earlier studies on driver mutations in MCV-negative tumors have succeeded in identifying prominent mutation. [9–13] While many mutational studies concerning MCC have been carried out in recent years, little is known about the expression of cancer genes at the RNA level.

In the current study, we aspired to examine the RNA expression of 50 known cancer genes in MCC tumors. We used targeted next-generation sequencing to assess the RNA expression profiles of both MCV-positive and MCV-negative tumors. The fundamental aim of our study is to find potential molecular targets to improve the treatment strategies of MCC.

## Methods

The Ethics Committee of Helsinki University Hospital approved the study and its plan. The Ministry of Health and Social Affairs granted permission to collect patient data, and the National Authority for Medicolegal Affairs to collect and analyze tissue samples.

From our pool of 270 formalin-fixed paraffin-embedded (FFPE) MCC tumor samples, we chose 13 MCV-negative and 13 MCV-positive tumors based on known MCV status and sufficient tumor sample available. MCC diagnoses were confirmed by morphology compatible with MCC in microscopy and by immunohistochemistry positive for CK-20 and negative for TTF-1. All tumor samples contained at least 50% of tumor tissue.

### Patients

Twenty-six patients with a primary MCC tumor were included in this study, 19 females and 7 males. The mean age of the patients was 79 years (range 59–100). The mean tumor size was 33 mm. The study group was divided into two subgroups based on the MCV status of the tumor samples. The MCV status was determined as described in our previous study. [14] Clinical data of the patients are presented in Table 1.

**Table 1:** Clinical data and ALK results

No	MCC^a^	MCV	age	Tumor location	Tumor size mm	ALK NGS^b^	ALK IHC D5F3 5A4	ALK FISH^c^
1	P2	pos	< 80	Right cheek		**+**	**+ −**	**−**
2	P3	pos	≥ 80	Posterior thigh	85	**+**	**+ −**	**+**
3	P4	pos	< 80	Thorax	70	**+**	**+ −**	**−**
4	P5	pos	< 80	Right knee	12	**+**	**+ −**	
5	P9	pos	≥ 80	unknown	20	**+**	**+ +**	**−**
6	N10	neg	≥ 80	Right arm	50	**+**	**+ +**	**+**
7	N11	neg	≥ 80	Left temple	15	**−**	**– –**	**−**
8	P12	pos	≥ 80	Forehead	40	**+**	**+ −**	**−**
9	P13	pos	< 80	Right buttock	34	**+**	**+ −**	**−**
10	P14	pos	≥ 80	Left cheek	18	**+**	**+ −**	**−**
11	N17	neg	< 80	Right cheek	20	**+**	**+ −**	**−**
12	N18	neg	< 80	Right breast	20	**+**	**+ −**	
13	N19	neg	< 80	Calf	13	**+**	**+ −**	
14	N21	neg	< 80	Neck	25	**+**	**+ −**	**–**
15	P23	pos	< 80	Left forearm	40	**+**	**+ −**	**−**
16	N25	neg	≥ 80	Left back		**+**	**+ −**	**+**
17	P26	pos	≥ 80	Right shoulder	24	**+**	**+ −**	**−**
18	P28	pos	≥ 80	Left arm	75	**+**	**−**	
19	N29	neg	≥ 80	Back	75	**−**	**– –**	**−**
20	N31	neg	< 80	Left foot	10	**+**	**+ +**	**−**
21	N32	neg	≥ 80	Right breast	23	**−**	(**+**) **–**	**−**
22	N33	neg	≥ 80	Front of left ear	18	**−**	(**+**) **–**	**−**
23	P34	pos	< 80	Flank	20	**+**	**+ +**	**+**
24	N35	neg	< 80	Upper abdomen	25	**+**	**+ −**	**−**
25	P36	pos	< 80	Right buttock	30	**+**	**−**	
26	N37	neg	≥ 80	Right cheek	30	**+**	**+ −**	**+**

^a^MCC tumor number. ^b^Cases that had high expression of *ALK* by NGS are marked +. ^c^Tumors with 3 or more dual signals in FISH are marked +. In cases N32 and N33, low proportion of tumor cells stained positive with D5F3 and thus are marked (+)

### RNA extraction

The total RNA was extracted from MCC tumor samples and two normal control skin samples. Extraction was performed according to the manufacturer’s manual using the miRNeasy mini Kit (Qiagen, Hilden, Germany). Qubit 2.0 Fluorometer (Thermo Fisher Scientific) and 2200 TapeStation System in combination with RNA ScreenTape assay (Agilent Technologies, Santa Clara, CA, USA) was used to measure the quantity and the quality of the RNA.

### Targeted next-generation sequencing

Quantitative RNA expression analysis was performed by amplicon-based next-generation sequencing (NGS) using Ion Torrent technology (Thermo Fisher Scientific, Waltham, MA, USA). The Ion AmpliSeq™ RNA Library Kit (Thermo Fisher Scientific, Waltham, MA, USA) was used to construct the libraries from 20 ng of RNA. RNA was reverse transcribed, and targeted regions of RNA were PCR amplified using the Ion AmpliSeq™ RNA Cancer Panel (Thermo Fisher Scientific, Waltham, MA, USA) consisting of specific primers sets to amplify 50 target genes. (Additional file 1: Table S1) The Amplicons were then partially digested, and barcode adapters were ligated with the Ion Xpress™ Barcode Adapter Kit (Thermo Fisher Scientific, Waltham, MA, USA) to yield a barcoded library. Library concentrations were measured using the Qubit 2.0 Fluorometer (Thermo Fisher Scientific, Waltham, MA, USA).

Templates for sequencing were prepared using the Ion PGM™ Hi-Q™ OT2 Kit (Thermo Fisher Scientific) and Ion OneTouch™ 2 System (Thermo Fisher Scientific, Waltham, MA, USA). Ion Sphere™ particles were enriched with Ion OneTouch ES (Thermo Fisher Scientific, Waltham, MA, USA) and loaded onto an Ion 318™ Chip (Thermo Fisher Scientific, Waltham, MA, USA).

Sequencing was performed on the Ion Torrent PGM™ System using the Ion PGM™ Hi-Q™ Sequencing Kit (Thermo Fisher Scientific, Waltham, MA, USA).

### Data analysis

Sequencing data was processed using Torrent Suite™ Software. The Coverage Analysis plugin was used to create amplicon counts. The mean length of amplicons was 150 bp. The amplicon count data created was imported into a Chipster [15] (http://chipster.csc.fi/index.shtml) for further differential gene expression analysis. Differential expression analysis, to compare expression differences between tumor and normal skin tissue and between MCV-positive and MCV negative tumors, was performed on read count data carried out with DESeq2. Differently expressed genes were determined from adjusted *p*-values and log^2^ fold change. To control for false positives, the *p*-values were corrected for multiple testing and the adjusted *p*-value or FDR (false discovery rate) calculated using Benjamini-Hochberg correction. An average of 344,458 reads (after quality check) were obtained for each sample with an average of 95% on target.

### ALK Immunohistochemistry

The sections (3 μm) were stained with fully automated immunostainer Ventana Benchmark XT (Roche/Ventana, Tucson, AZ, USA). For both ALK antibodies we used heat- induced epitope retrieval buffer Cell Conditioning 1 (Roche/Ventana, 950–124) for 64 min in 98 °C. The dilutions and incubation times for ALK antibodies were: clone 5A4 (Novocastra™, Leica Biosystems, Wetzlar, Germany) 1:100 for 40 min/36 °C and clone D5F3 (Ventana/Roche, Tucson, AZ, USA) 28 min/36 °C. The three- step, multimer based detection kit, OptiView (760–700, Roche/Ventana), was used to detect the antibodies. Amplification step was added for both protocols by using separated amplification kit (Roche/Ventana, 760–099). The slides were finally stained with hematoxylin (Mayer, S3099, Dako, Glostrup, Denmark). For the control of the staining quality we used skin, appendix and known ALK positive and ALK negative tumor tissue. The stained slides were examined by researchers TB, TV, MK and VKS.

### Fluorescence in situ hybridization (FISH)

FISH was performed on 2 μm thick FFPE tumor sections. The sections fixed on microscopic slides were de-paraffinized, pre-treated with protease and hybridized with Vysis LSI ALK Dual Color Break Apart FISH probes according to the vendor’s guidelines (Abbott Molecular Inc., Des Plaines, IL, USA) and as described previously. [16] Results were checked under a fluorescence microscope. The criteria for considering a cell to be *ALK* gene rearrangement positive was: presence of at least one green and orange signal pair split apart by ≥2 signal diameters (pair-signal type fusion), or a single orange without corresponding green signal (single-signal type fusion). The cells were considered *ALK* fusion negative if they had fused or if they had close orange and green signal.

## Results

### MCC vs non-cancerous tissue

**Fig. 1:**
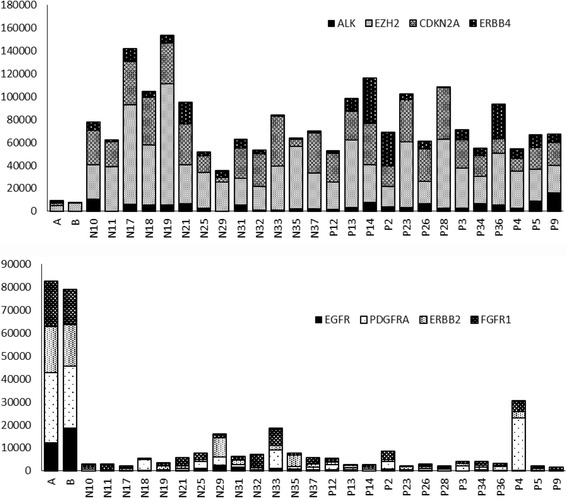
Differently expressed genes in MCC tumors (N: MCV-negative, P: MCV-positive) compared to normal skin tissue (A and B). *Upper panel* shows normalized expression of all statistically overexpressed genes (*ALK, EZH2, CDKN2A* and *ERBB*) and *lower panel* shows expression of all underexpressed genes *(EGFR, PDGFRA, ERBB2* and *FGFR1*) in MCC compared with normal skin

Gene expression of all 26 MCC tumor samples compared to that of normal skin samples showed eight significantly (*p*-value <0.005 and log2 fold change of at least 2) differently expressed genes by DEseq2 (Table 2). Overexpression of *ALK, CDKN2A*, *EZH2* and *ERBB4* was recorded in MCC samples compared with normal skin samples. Downregulation of *EGFR*, *ERBB2*, *PDGFRA* and *FGFR1* was evident in MCC tumors compared with normal skin samples (Fig. 1).

**Table 2:** RNAs with differential expression in MCC tumors compared with normal skin as analyzed by DESeq2

Gene	log2FoldChange	*p*-value	adjusted *p*-value
*ALK*	7,6	1,21E-16	6,03E-15
*CDKN2A*	4,4	1,10E-11	2,74E-10
*EZH2*	2,7	2,45E-07	4,08E-06
*EGFR*	-4,8	2,56E-06	3,20E-05
*ERBB2*	-3,7	6,10E-05	0,0006104
*FGFR1*	-2,9	0,0002632	0,002194
*PDGFRA*	-3,6	0,0007482	0,005344
*ERBB4*	2,5	0,006786	0,04241

**Fig. 2:**
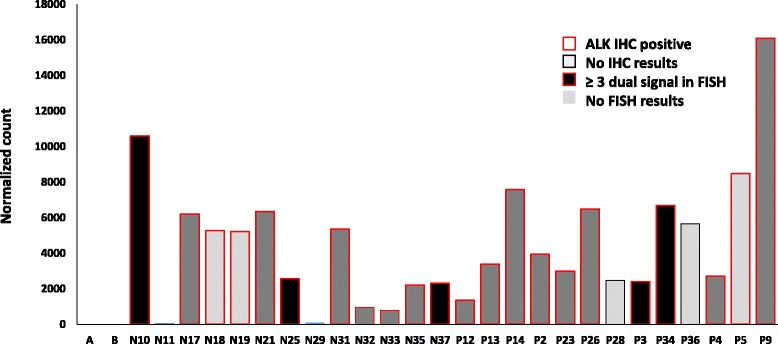
Normalized RNA expression of *ALK*, its correlation with its protein expression as studied by immunohistochemistry (Clone D5F3) and DNA alterations (copy number) as seen by FISH in MCC tumors (N: MCV-negative, P: MCV-positive) and normal skin (A and B)

**Fig. 3:**
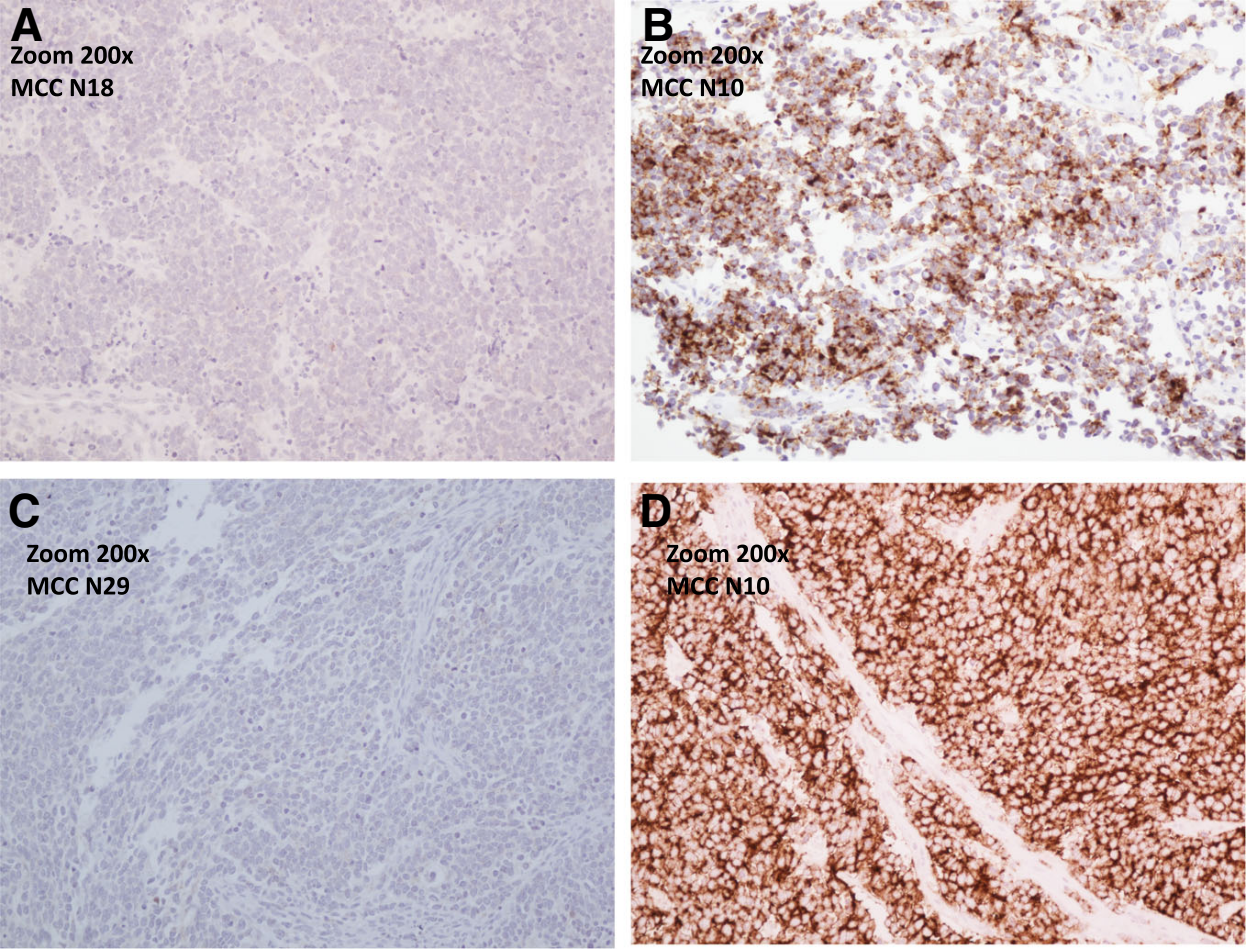
ALK Immunohistochemistry in MCC tumor samples. **a**: negative ALK IHC (Clone 5A4) in MCC tumor sample (Sample N18, 200× magnification). **b**: positive ALK IHC (Clone 5A4) in MCC tumor sample (Sample N10, 200× magnification). **c**: negative ALK IHC (clone D5F3) in MCC tumor sample (Sample N29, 200× magnification). **d**: positive ALK IHC (Clone D5F3) in MCC tumor sample (Sample N10, 200× magnification)

*ALK* was the most significantly overexpressed gene in tumor tissue of MCC patients, with a log2fold change of 7.6. *ALK* expression was absent or very low in normal skin; a normalized read count of 0 and 19 in normal skin compared to a mean normalized read count of 4549 (range 71–16,090; >1000 in 22/26) in tumor samples (Fig. 2). In order to see whether ALK was also expressed at protein level, we performed immunohistochemistry on tumor and normal skin tissue sections using two different antibodies. We also performed FISH on sections to investigate whether increased expression was related to any genetic alterations involving *ALK* (Fig. 2)*.* ALK immunohistochemistry was positive in 22 MCC tumors with antibody clone D5F3 and in four tumors with clone 5A4**.** All ALK positive tumor samples by IHC also showed high RNA expression. Both the normal skin samples and normal skin around the tumors were negative for ALK. IHC stainings are illustrated in Fig. 3.

### Fluorescence in situ hybridization

FISH results were obtained from 21 tumor samples and 2 normal skin samples. For the remaining tumors, the signals were too weak to get any reliable results, and thus were not included in the FISH analysis.

We did not find any break-apart signals in any of the tumor samples, thereby ruling out the possibility of translocation/inversion. However, in five samples, three or more dual signals were seen in local regions indicating gain/polyploidy. These five samples stained positive for ALK in IHC with clone D5F3 and two of these samples were also positive with clone 5A3. *ALK* expression by NGS, IHC and FISH results are presented with the clinical data in Table 1.

### MCV-negative and -positive tumors

**Fig. 4:**
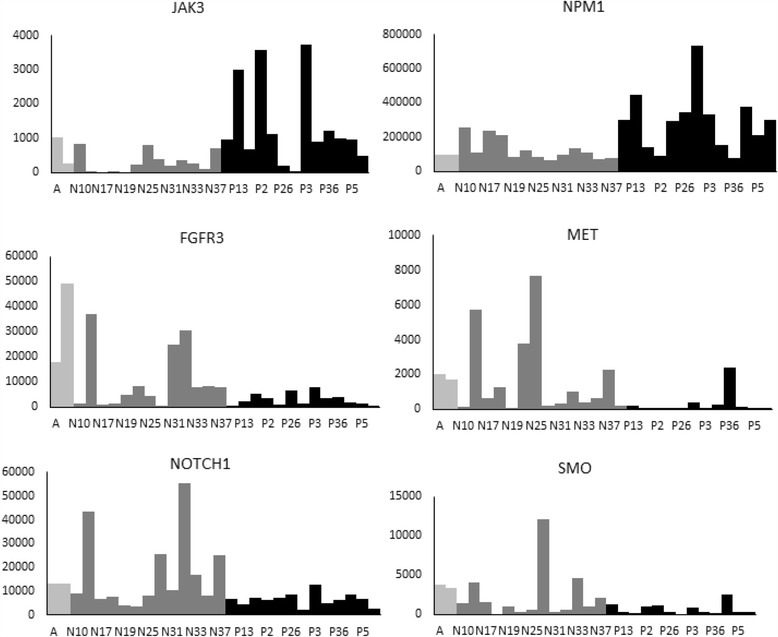
Normalized expression of genes differently expressed between MCV-negative and MCV-positive tumors. *Light grey*: normal skin tissue; *dark grey*: MCV-negative tumors (N); *black*: MCV-positive tumors (P)

Hierarchical clustering did not separate the MCV-negative from the MCV-positive group based on the expression of the 50 genes. However, six genes showed statistically differential expression between the MCV- positive and - negative groups (Table 3, Fig. 4). In the MCV-negative tumors, *MET, NOTCH1, FGFR3, and SMO* were overexpressed and *JAK3* and *NPM1* were under-expressed compared to the MCV-positive tumors.

**Table 3:** RNAs with differential expression in MCV-negative tumors compared to MCV-positive as analyzed by Deseq

deseq NvsP	log2FoldChange	*p*-value	adjusted *p*-value
NOTCH1	1,3	9,17E-05	4,58E-03
NPM1	-1,1	2,35E-04	5,88E-03
FGFR3	1,6	3,97E-04	6,62E-03
MET	1,9	7,59E-04	9,49E-03
JAK3	-1,6	3,03E-03	3,03E-02
SMO	1,5	6,38E-03	4,56E-02

## Discussion

In this study, we analyzed the RNA expression of 50 cancer-related genes in 26 MCC tumors by targeted next-generation sequencing and compared their expression with normal, non-cancerous skin samples. Among the 50 cancer-related genes, we identified eight genes (Table 2) that had differential expression in tumor tissue. Further confirmation of the results with quantitative PCR in larger tumor cohort and comprising more genes is required.

We recorded overexpression of cancer related genes including *ALK*, *CDKN2A,* and *EZH2* compared with normal skin. Among the under-expressed genes, we identified *EGFR,* in concordance with our earlier study showing negative EGFR expression by immunohistochemistry in MCC tumors. [9] Earlier studies have found inactivating *RB1* and *TP53* mutations driving MCV-negative tumors, [10] however we did not find different expression of *RB1* or *TP53* in MCV-negative tumors compared to MCV-positive tumors.

In this study, we recorded for the first time *ALK* overexpression in tumors of MCC patients. *ALK* expression was seen in all patients with high levels in 22/26 tumor samples (Table 1, Fig. 2). *ALK* overexpression at the mRNA level seen in our results fits well with the protein expression results of the only ALK IHC study on MCC [17], whereby they demonstrated ALK expression in 30/32 MCC tumors by one (clone D5F3) of the three antibodies used in the study. They however noted that the frequency of ALK positive cases for MCC tumors depended a lot on the antibody used and found ALK positivity in 4/32 with the ALK1 clone and 28/32 with clone 5A4. In our IHC analysis we used the clones D5F3 and 5A4. Positive results were seen in 22/24 (Clone D5F3) and 4/26 (Clone 5A4) tumors, and only in those that had a high RNA expression. IHC staining was more even and vivid with clone D5F3 (Fig. 3). Therefore, our results correspond well with the previous ALK IHC study and it seems that clone D5F3 is the most sensitive clone in detecting ALK expression in MCC tumors.

ALK is normally expressed predominantly in central nervous system and it likely functions in development of the brain. ALK is however, known mainly for its role in various types of cancer. One common mechanism of ALK activation in tumors is *ALK* gene rearrangement leading to fusion protein like NPM-ALK in anaplastic large cell lymphomas [18] and EML4–ALK in non-small-cell lung cancer. [19]. In order to study whether the mechanism behind the mRNA overexpression is a fusion gene or gene amplification, we performed the FISH analysis. As no fusions or high level amplification (only 2–3 times gain in 5 cases) were seen by FISH, the mechanism behind *ALK* overexpression might possibly be epigenetic or as a result of over-activation of a transcription factor. Similar to our results, no rearrangement or other cytogenetic aberration of the *ALK* gene have been reported in MCC. [17].

ALK expression without any rearrangement or amplifications of the *ALK* has, been reported in hepatocellular carcinoma associated with poor prognosis and occurrence of micrometastases [20]. While no clear driving pathway in MCC has been identified, ALK is expressed in MCC tumors at both the RNA and protein level, and therefore, studying the mechanism and significance of this overexpression remains intriguing. Studying the correlation between ALK expression and survival in a larger MCC tumor cohort would be of future interest.

*CDKN2A* was another gene overexpressed in MCC tumors compared to normal skin. Our results are in concordance with our group’s previous study showing an expression of p16 (encoded by *CDKN2A*) by IHC in 97.7% of 88 MCC tumors. [21] *CDKN2A* is frequently mutated or deleted in a wide variety of tumors, including malignant melanoma and MCC and is considered to be a tumor suppressor gene. [9, 22, 23] p16 overexpression in malignant tumors is thought to be a mechanism to overcome proliferation resulting from the failure of the RB1 pathway due to viral infection, genetic/epigenetic silencing of *RB1* gene or other mechanism. [24] p16 overexpression is additionally reported in HPV infection associated cervical dysplasia and carcinoma as well as in cervical neuroendocrine tumors. [25–27] In MCC, *CDKN2A* RNA expression and p16 protein expression were, however, independent from the tumors’ MCV-status, although the role of *RB1* in MCC is reported earlier. [28].

The expression profile of MCC tumors also showed overexpression of *EZH2* that codes for an enzyme important in heterochromatin formation via DNA methylation. Mutations or overexpression of *EZH2* are seen in many forms of malignancies. [29, 30] It is thought that overexpression of EZH2 inhibits the transcription of tumor suppressors, thus promoting malignant transformation. Inhibitors of EZH2 are in development, and their role in cancer treatment is being studied. In malignant melanoma, Zingg et al. recently found that EZH2 expression correlated with poor survival and promotes the initiation and progression of melanoma in mouse models as well as human cell cultures. Using both an RNA interference and preclinical drug GSK305 to target EZH2, they showed that EZH2 could be a promising therapeutic target in the treatment of melanoma patients. [31] To our knowledge, *EZH2* expression is not previously reported in MCC tumors. It could be that EZH2 transcriptionally silences important tumor suppressors in MCC, similarly to its function in melanoma. Studying the role of the overexpression of *EZH2* in MCC oncogenesis would be of interest as a drug target.

Comparison of gene expression among the MCV –negative and positive groups showed a higher expression of *MET, SMO, FGFR3 and NOTCH1* in MCV-negative tumors compared to MCV positive tumors. *MET* is a known target of microRNA-34a, and in our previous study, we had reported under-expression of this miRNA in MCV-negative MCC. [7]. Notably, two genes, *JAK3* and *NPM1* were overexpressed in MCV-positive tumors compared to MCV-negative. *JAK3* is predominantly expressed in immune cells. To our knowledge, *JAK3* mutations have not been reported in MCC. Activating *JAK3* mutations are seen in hematological malignancies. Deficiency/defects in *JAK3* leading to low amounts of functional protein are associated with immune dysfunction/immunodeficiency. [32] *NPM1* is involved in many processes and is a fusion partner with many genes, especially *ALK*. Overexpression of *NPM1* is reported in many tumors including HCC, colon cancer and glioblastoma. [33–36]. *NPM1* associates with viral proteins of different viruses and is implicated in various stages of viral infection. [37], and this might be a reason for its higher levels seen in MCV-positive tumors. The significance of the genes differentially expressed between the MCV-negative and positive groups is however not clear and needs further investigation.

## Conclusions

We established that *ALK* is overexpressed in MCC tumors, although no ALK fusion was seen by FISH analysis. Thus significance of ALK in MCC remains uncharted, yet intriguing. Overexpression of *EZH2* suggests its potential as a drug target in MCC.

## Additional files

Additional file 1: Table S1. Ion AmpliSeq™ RNA Cancer Panel target genes. The table presents the 50 genes that were investigated in this study. (DOCX 12 kb)

Additional file 2: Table S2. The normalized RNA expression data. The table presents the normalized RNA expression data recorded in this study. (XLSX 30 kb)

## Abbreviations

MCC: Merkel cell carcinoma
MCV: Merkel cell polyomavirus
FFPE: Formalin-fixed paraffin-embedded
FISH: Fluorescence in situ hybridization
NGS: Next-generation sequencing
IHC: immunohistochemistry
HPV: Human papillomavirus
HCC: Hepatocellular carcinoma
