# Supplementary material for: Aberrant expression of ALK and EZH2 in Merkel cell carcinoma
Source: BMC Cancer. 2017 Mar 31;17:236. doi: 10.1186/s12885-017-3233-5 (PMC5374569; doi:10.1186/s12885-017-3233-5)
Supplement: Supplementary file 1 — Ion AmpliSeq™ RNA Cancer Panel target genes. The table presents the 50 genes that were investigated in this study. (DOCX 12 kb) [file 12885_2017_3233_MOESM1_ESM.docx]

| *ABL1* | *CSF1R* | *FGFR2* | *IDH1* | *MLH1* | *PTPN11* | *TP53* |
| --- | --- | --- | --- | --- | --- | --- |
| *AKT1* | *CTNNB1* | *FGFR3* | *JAK2* | *MPL* | *RB1* | *VHL* |
| *ALK* | *EGFR* | *FLT3* | *JAK3* | *NOTCH1* | *RET* |  |
| *APC* | *ERBB2* | *GNA11* | *IDH2* | *NPM1* | *SMAD4* |  |
| *ATM* | *ERBB4* | *GNAS* | *KDR* | *NRAS* | *SMARCB1* |  |
| *BRAF* | *EZH2* | *GNAQ* | *KIT* | *PDGFRA* | *SMO* |  |
| *CDH1* | *FBXW7* | *HNF1A* | *KRAS* | *PIK3CA* | *SRC* |  |
| *CDKN2A* | *FGFR1* | *HRAS* | *MET* | *PTEN* | *STK11* |  |

| **Supplementary Table 1**. Ion AmpliSeq™ RNA Cancer Panel target genes. |
| --- |
